# Inferring Molecular Processes Heterogeneity from Transcriptional Data

**DOI:** 10.1155/2017/6961786

**Published:** 2017-12-06

**Authors:** Krzysztof Gogolewski, Weronika Wronowska, Agnieszka Lech, Bogdan Lesyng, Anna Gambin

**Affiliations:** ^1^Institute of Informatics, University of Warsaw, Banacha 2, 02-097 Warsaw, Poland; ^2^Faculty of Biology, University of Warsaw, Miecznikowa 1, 02-096 Warsaw, Poland; ^3^College of Inter-Faculty Individual Studies in Mathematics and Natural Sciences, University of Warsaw, Banacha 2c, 02-097 Warsaw, Poland; ^4^Bioinformatics Laboratory, Mossakowski Medical Research Centre, Polish Academy of Sciences, Pawińskiego 5, 02-106 Warsaw, Poland

## Abstract

RNA microarrays and RNA-seq are nowadays standard technologies to study the transcriptional activity of cells. Most studies focus on tracking transcriptional changes caused by specific experimental conditions. Information referring to genes up- and downregulation is evaluated analyzing the behaviour of relatively large population of cells by averaging its properties. However, even assuming perfect sample homogeneity, different subpopulations of cells can exhibit diverse transcriptomic profiles, as they may follow different regulatory/signaling pathways. The purpose of this study is to provide a novel methodological scheme to account for possible internal, functional heterogeneity in homogeneous cell lines, including cancer ones. We propose a novel computational method to infer the proportion between subpopulations of cells that manifest various functional behaviour in a given sample. Our method was validated using two datasets from RNA microarray experiments. Both experiments aimed to examine cell viability in specific experimental conditions. The presented methodology can be easily extended to RNA-seq data as well as other molecular processes. Moreover, it complements standard tools to indicate most important networks from transcriptomic data and in particular could be useful in the analysis of cancer cell lines affected by biologically active compounds or drugs.

## 1. Introduction

RNA microarrays and RNA-seq are one of the most popular high-throughput methods used in the advanced medical diagnostics, personalized medicine, and basic research. Although an application of these methods provides an insight into the full transcriptome of examined sample, the knowledge gained in this way is based upon an averaged gene expression in a bulk population. This fact introduces a specific bias into the outcome of gene expression measurements, especially, because a biological material is rarely homogeneous. A sample heterogeneity is either due to the diversity of cell types in case of tissue samples, or due to minor gene expression differences in samples obtained from cell lines. In both cases an observed average expression level may conceal relevant, cell-specific properties or mechanisms activated only in subpopulations of cells [1, 2]. Therefore, we have developed a novel computational method to infer the contribution of cell subpopulations to the observed expression of genes. We call the proposed method MPH after Molecular Process Heterogeneity. So far, a couple of methods were proposed to deal with the problem of mixed cell types in biological samples, that is, tissues. Mostly they are based on the expression matrix decomposition and yield the information about (i) proportions of different cell types in a given sample and (ii) expression profiles specific for each detected cell-type. As an example, in [3], authors introduce the method based on the least squares nonnegative matrix factorization for discovery of cell-specific marker genes with noisy signals because of varying cell-type proportions in a sample. The state of the art in computational methods for determination of sample cellular content and cell-specific expression profiles is summarized in [4]. However, not only the subpopulation-specific behaviour, but also limited information about gene regulatory networks reduces the possibility of a meaningful inference from the transcriptomic data.

A closely related topic refers to the reconstruction of gene regulatory networks from mRNA expression data. A plethora of methods were proposed, but none of them brought a spectacular success. In particular, Zhang et al. [5] presented a method considering the path consistency algorithm based on the conditional mutual information. Also some improvements of the standard path consistency algorithms have already been proposed, such as the elimination of the gene ordering problem [6]. The other approach proposed by Dojer et al. [7] successfully applies dynamic Bayesian networks for the gene regulatory network inference based on the perturbed gene expression data.

Here, we decided to explore the already existing knowledge on the regulome and the signalome to provide an insight into the heterogeneity of molecular processes in a cell population under study. Therefore, the proposed methodology complements the above-mentioned procedures for inferring gene regulatory networks. Our method explores the functional heterogeneity of a given cell population sample through the quantification of the intensity of molecular processes occurring in it.

The proposed approach was tested using the data from two independent* in vitro* experiments. The first one was aimed to assess the global transcriptomic changes occurring in the ovarian cancer cell line SKOV3 upon paracrine signaling present in the peritoneal cavity. The second one was aimed to decipher how the ceramide induced pathways are affected by the inhibition of poly(ADP-ribose) polymerase (PARP) in the neuroblastoma cell line SH-SY5Y. In both test-case studies the MPH method was used on microarray expression data to estimate the changes in the viability of variously treated populations of cells. The obtained computational predictions were validated with the results of additional wet-lab assays.

The paper is organized as follows. In the next section we present the workflow of the MPH method and validation strategies that are applied. Materials and Methods contains the description of the biological experiments and the detailed presentation of the computational method. In Results an insight into the final outcomes from the MPH method is provided and in Discussion we elaborate about the compatibility of computational and experimental results as well as the method effectiveness. Finally, in Conclusions we summarize our work and point out the future steps that we plan to undertake.

## 2. Overview of the MPH Method

Our goal is to estimate the proportion of different cell subpopulations existing in the analyzed sample being a type-homogeneous population of cells. We assume that there exist at least two different molecular processes manifested by different transcriptional activity of a specific cell subpopulation. Specifically, the proportion should be understood as a qualitative contribution of each subpopulation into the transcriptomic signal observed in the data retrieved from the whole sample.

Along with the proportions, we determine transcription patterns specific for each subpopulation. Again, it should be mentioned that values assigned to the expression of each gene in the patterns are not strictly levels of transcription, but rather correspond to the trends observed in detected subpopulations. The MPH method described below was validated using the gene expression data from two case studies described in detail in Materials and Methods.

**Figure 1:**
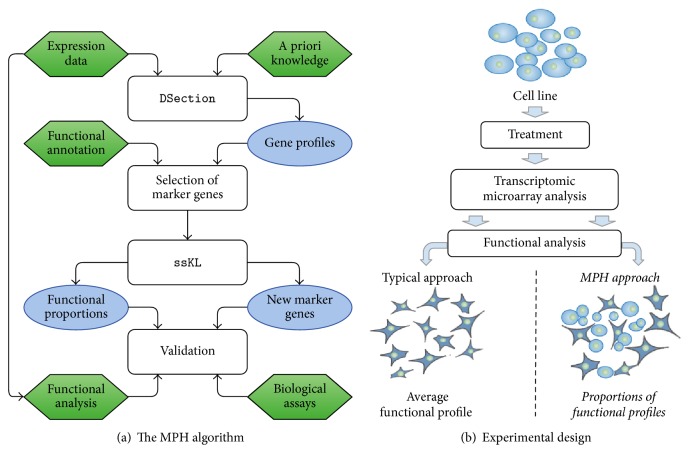
An overview of the concept of the article: (a) an insight into the workflow of the MPH algorithm; (b) the general use case of our algorithm.

To quantify the composition of the sample, we adapted the computational framework designed originally for the deconvolution of a gene expression matrix from heterogeneous samples [8]. The method decomposes an expression matrix into components representing the description of different tissues that were mixed in the sample. Here, since we assume the sample homogeneity (as we study the cell lines), the deconvolution is expected to reveal the heterogeneity at molecular processes level; that is, different expression profiles are inferred for cell subpopulations that proliferate and those that remain in a dormant state (case study 1) or proapoptotic condition (case study 2).  Figure 1 presents the outline of the MPH method.

The procedure starts with the routine processing of the raw data from the microarray experiments resulting in normalized, filtered (i.e., quality controlled) expressions for each experimental scenario. Then, for the further analysis we select only these genes that differentiate the experimental conditions in the considered study in a statistically significant manner.

The next step is the DSection algorithm (i.e., an unsupervised matrix decomposition method) [9]. It requires the starting proportions as a priori knowledge for Bayesian model and provides information about specific gene profiles detected during the decomposition of the expression matrix. Intuitively, one can think of this step as the form of a clustering procedure; the algorithm points out samples described by similar transcription patterns and consequently by potentially similar functional activity, given the expected proportion, which can be randomly or uniformly distributed if no additional knowledge is provided. From these gene profiles statistically significant genes are selected, then annotated, and validated using DAVID tool [10], to finally constitute a marker list per profile describing specific functions and pathways currently active in the cells under the study.

The lists of marker genes are used as an input to the second decomposition phase called ssKL which is a modified version of the algorithm proposed in [11]. The outcome of this step is twofold: the estimated proportions of functionally homogeneous cell subpopulations and the gene expression profiles specific for these subpopulations, thus also for the molecular processes taking place in them.

Results of this step are validated using the dedicated* in vitro* wet-lab assay (in our case studies the Resazurin Reduction Assay and the MTT assay, resp.). Finally, marker genes from the previous step were assessed to find out whether they characterize specific functions.

The MPH framework was used to verify two hypotheses that were addressed in the conducted experiments. First, we want to assess and compare the putative paracrine influence of K21 fibroblasts and SKOV3 cells on ovarian cancer cell proliferation. It is presumed that, despite serum-free conditions, secretion products of both cell types are able to prompt SKOV3 cell divisions and that the stromal signaling exerts a stronger effect.

In the second case, we want to computationally quantify the influence of PJ34-PARP inhibitor on the viability of the C2-ceramide treated cells. The hypothesis claims that PJ34 poses a cytoprotective character. For detailed description of the biological background of both case studies see Appendix A.

Finally, thanks to our approach that determines function-specific transcriptomic profiles, we provide the most important markers characterizing concrete cellular functions. Here, it should be emphasized that the knowledge about gene regulation in cancerous cells is still quite vague and selection of novel biomarkers for specific cellular processes might improve our understanding of cancer development mechanisms.

## 3. Materials and Methods

### 3.1. Cell Culture Experiments

The ovarian cancer cell lines SKOV3 (HTB-77) and the human neuroblastoma cell line SH-SY5Y were obtained from American Type Culture Collection (ATCC); healthy fibroblast cell line K21 was a gift from Dr. Barbara Tudek (Institute of Genetics and Biotechnology, Polish Academy of Science). Cells were maintained at 37°C in a humidified incubator containing 5% CO_2_.

*Case Study 1*. SKOV3 and K21 cells were cultivated in a high glutamine RPMI (Cytogen), supplemented with 10% FBS (Cytogen), 20 mM HEPES (Cytogen), and 1% Penicillin-Streptomycin solution (Cytogen). To obtain conditioned media, cells were cultivated up to 80% confluence, washed three times with PBS solution, and incubated in the serum-free culture media. Following the 72-hour growth, the conditioned media (CM) were collected and filtered (0,22 um) to remove cellular debris.

*Case Study 2*. The SH-SY5Y (Sigma-Aldrich) cells were cultured in MEM/F-12 Ham Nutrient Mixtures (1 : 1) (Biowest) supplemented with 15% heat-inactivated FBS (Cytogen), 1% penicillin/streptomycin, and 2 mM glutamine (Sigma-Aldrich). Prior to treatment, the cells were cultivated in low serum medium (2% FBS).

### 3.2. Microarray Experiments

*Case Study 1*. For the microarray experiments, SKOV3 cells were cultured up to 70% confluence, washed twice with PBS, and incubated for 48 hours in media conditioned by SKOV3 cells (CM-SKOV3) or K21 (CM-K21). Following CM culture, mRNA was isolated using Trizol reagent followed by DNase treatment (Roche) and purification using RNAeasy extraction kit from Qiagen. Samples of 200 ng of total RNA were analyzed on Affymetrix GeneChip Human Genome U133 Plus 2.0 Array. We analyzed three RNA samples per condition.

*Case Study 2*. SH-SY5Y cells were cultivated for 3, 6, and 24 hours in the following experimental settings supplied with (i) 25 *μ*M C2-ceramide d18:1/2:0 (Enzo Life Sciences); (ii) 25 *μ*M C2-eramide d18:1/2:0 (Enzo Life Sciences) and 20 *μ*M PARP-1 inhibitor PJ-34 (Sigma-Aldrich); (iii) pure medium for control samples. The experiment was done in three replicates. After 3, 6, and 24 hours of cultivation cells were collected and RNA was purified using Affymetrix PrepEase RNA Spin Kit. The whole-transcript analysis was conducted using Affymetrix Human Gene 2.1 ST Array Plates.

Gene lists were annotated and analyzed using Qiagen's Ingenuity Pathway Analysis tools (https://www.qiagen.com/ingenuity, IPA®, Qiagen Redwood City).

### 3.3. Cell Proliferation Assay

*Case Study 1*. Cell proliferation was performed in six repeats by Resazurin (Sigma-Aldrich) Reduction Assay. Briefly, 2000 SKOV3s/well were seeded in 96-well plate. Following treatment for the indicated time, 10 *μ*l of 0,01% resazurin solution was added to each well and incubated at 37°C for up to 72 h. The fluorescence was measured at different time points with a Multimode Microplate Readers (BioTek) at a wavelength of 590 nm. Proliferation curves were analyzed using GraphPad Prism software (version 5, GraphPad Software, Inc.).

*Case Study 2*. After treating SH-SY5Y cells with different compounds, cell viability was evaluated using 2-(4,5-dimethylthiazol-2-yl)-2,5-diphenyltetrazolium bromide assay (MTT; Thermo Fisher Scientific). Representative samples were collected from each experimental setting, MTT was added to all of the wells. The cells were incubated at 37°C for 2 h, followed by cell lysis and spectrophotometric measurement at 595 nm.

### 3.4. Computational Method

In our work we applied the matrix deconvolution algorithms that originally were used in the analysis of heterogeneous samples being composed of different cell types in some proportions. In general, these methods cover a wide range of approaches which try to decompose a gene expression matrix *X* (rows represent genes; columns represent samples) into the product of two matrices *W* and *H* of given rank *k* (the rank corresponds to the number of expected cell types in a sample):

(1) $$\begin{array}{c} X=WH+\epsilon , \end{array}$$

where *W* is a matrix with a cell-specific transcriptome (rows represent genes and each column corresponds to one of the expected cell types), *H* is a matrix with proportions of cells in each sample (rows represent the expected cell types; columns represent samples), and *ϵ* models the error/noise.

In this study we are especially interested in two algorithms: DSection and ssKL. DSection algorithm [9] is an unsupervised approach to the matrix decomposition problem. The only knowledge, that is expected as an input, is the initial, a priori proportion of cell types. The algorithm estimates both cell-specific transcriptome and cell proportions using the Markov Chain Monte Carlo approach.

In the context of our problem, assume we have *J* cell-type homogeneous samples; in each sample *T* cell subpopulations are involved in different molecular processes and *C* experimental conditions. For each sample *j* the a priori knowledge **p**_*j*_ = (*p*_1*j*_,…, *p*_*Tj*_) about proportions of cells with specific function is also given. Then the expression *y*_*ij*_ of the *i*th gene in *j*th sample is described as

(2) $$\begin{array}{c} y_{i,j}=p_{j}x_{i,c\left(j\right)}=\overset{T}{\underset{t=1}{\sum }}p_{t,j}x_{t,i,c\left(j\right)}+\epsilon_{i,j}, \end{array}$$

where **x**_*i*,*c*(*j*)_ = (*x*_1,*i*,*c*(*j*)_,…, *x*_*T*,*i*,*c*(*j*)_) represents the contribution of different cell subpopulations (i.e., different molecular activities of cells) to the observed *i*th expression profile and *ϵ*_*i*,*j*_ is a normally distributed noise reflecting replication noise with variance 1/*λ*_*i*_, for some constant *λ*_*i*_. Hence, the likelihood of *y*_*i*,*j*_ can be described as

(3) $$\begin{array}{c} y_{i,j}\;|\;p_{j},x_{i},\lambda_{i}\sim N\left(\;\overset{T}{\underset{t=1}{\sum }}p_{t,j}x_{t,i,c\left(j\right)},\frac{1}{\lambda_{i}}\;\right). \end{array}$$

Note that the replication variance, 1/*λ*_*i*_, is heteroscedastic across probes and homoscedastic across cell subpopulations and experimental conditions. Finally, assuming i.i.d. measurements (which may not always be the case) a factorized form for the joint data (*𝒟*) likelihood can be written as

(4) $$\begin{array}{c} f\left(\;D\;|\;\theta \;\right)=\overset{I}{\underset{i=1}{\prod }}\overset{J}{\underset{j=1}{\prod }}f\left(\;y_{i,j}\;|\;p_{j},x_{j},\lambda_{i}\;\right), \end{array}$$

where *θ* represents all model parameters; that is, *θ* = (**p**, **x**, *λ*). More detailed description of prior specifications, sampler construction, and posterior sampling, that is, probability of acceptance of one step in constructed mixed Gibbs and Metropolis-Hastings sampler, can be found in [9].

On the other hand, ssKL is an approach that is based on iteratively computed approximation of a desired decomposition. In our case it is supported by a list of marker genes characterizing molecular functions that are active in the sample of interest and regulate its observed transcriptome. Specifically, the factorization *X* ~ *WH*, given a desired rank *k* (i.e., number of expected cell subpopulations/activities), starts with a random initialization of *W* and *H* matrices which are then updated to minimize a divergence functional:

(5) FX,W,H=∑i,jXi,jlog⁡Xi,jWHi,j−Xi,j+WHi,j.

The approximation in the algorithm is performed in two steps using the coupled divergence equations:

(6) Hi,j⟵Hi,j∑tWt,iXt,j/WHt,j∑kWk,i,Wt,i⟵Wt,i∑jHi,jXt,j/WHt,j∑lHi,l.

It should be emphasized that even though the algorithm does not need to necessarily converge to the same solution on each run (see [11] for details), in our case study the method turns out to provide consistent and robust results throughout repetitive runs.

Both presented algorithms originally referred to the identification of cell-type specific transcriptomic profiles composing the investigated sample. However, in the next section we will show that these methods can be successfully applied to determine the activity-related proportions and markers in cell-type homogeneous samples that are characterized by the heterogeneity of undergoing molecular processes.

## 4. Results

Here, we assume that the expression level for each gene is the result of an expression activity in two distinct cell subpopulations. In our case studies one of them consists of actively dividing cells and the second one consists of cells with activated specific signaling pathways of the other type. Building on this premise, the decomposition of the expression matrix yields the proportions of the characterized cell subpopulations.

### 4.1. Marker Genes Selection

In order to assure the computational efficiency of used algorithmic solutions, we first reduce the number of analyzed genes. We use only genes that differentiate experimental conditions in a statistically significant way according to the two-sample *T*-test with *p* value less than 0.05. Such filtering resulted in 3946 and 3983 genes, respectively, per case study, that were used in further decomposition analysis.

According to the presented workflow, we first answered the question if samples' profiles for such selected genes exhibit any specific inner structure. Application of DSection algorithm determined a decomposition of the expression matrix. Taking into consideration the character of our experimental data and provided assays, in both our case studies we set the prior knowledge as a uniform proportion of two functional subpopulations.

In each case study, annotation of the genes that differentiate well between two obtained profiles (i.e., top 500 genes with the highest fold change) was performed using DAVID tool [10, 12]. It provides groups of marker genes characteristic for each profile based on the functional enrichment analysis. This classification was systematically verified using literature reports on each gene function. For the subsequent analysis only genes known to enhance particular process were selected.

In the first case study we end up with the marker genes for (i) the proliferation activity composed of 12 genes, CDC20, TK1, KNL1, CENPE, STIL, ANLN, NDC1, NUF2, KIF20A, PLK4, CCNB1, and CCNA2, and (ii) the quiescence state composed of 12 genes: COL5A1, TGFBI, TCEA2, WNT9A, MMP11, LAMB1, KRT14, LTBP1, PHLDB1, TIMP3, LRP1, and COL18A1.

On the other hand, in the second case the following marker genes are described: (i) proliferation regulation including 11 genes, CD24, NRP1, TNS3, MYC, CD38, CCNA2, FGF7, MST1R, MYCN, ETS1, and EDNRA, and (ii) cell death related regulation including 12 genes: DDIT3, ERN1, JUN, SQSTM1, SMPD1, TGFB1, PRNP, CEBPB, NQO1, NR4A1, CTSB, and ZMAT3.

**Figure 2:**
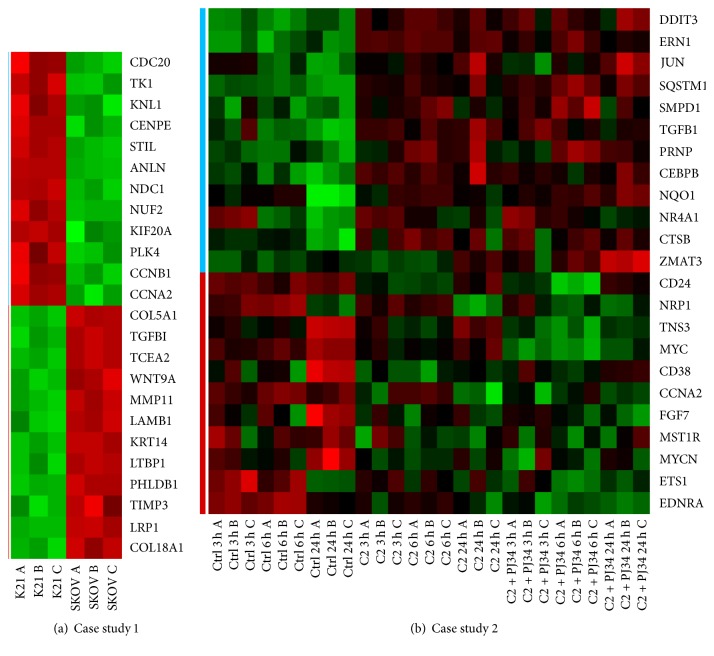
Expression patterns of marker genes for each case study. The (a) panel describes 12 genes that regulate proliferation and 12 genes that regulate maintenance/survival functions. On the other hand, the (b) panel describes 12 genes related to positive regulation of the cell death processes (blue stripe) and 11 genes related to positive regulation of proliferation mechanisms (red stripe).

The expression patterns of all selected marker genes can be investigated in Figure 2, while their full names are given in Appendix B (Tables 3 and 4). Additionally, we have performed a bunch of tests to detect how the outcome of the MPH method is dependent on the number of marker genes used in the ssKL step. It turned out that a set of 10–12 genes is sufficient to keep the percentage results robust. Moreover, we verified how the average fold change of selected marker genes influences the final predicted proportions in the ssKL algorithm; see Appendix C.

### 4.2. Estimation of Functional Heterogeneity

**Figure 3:**
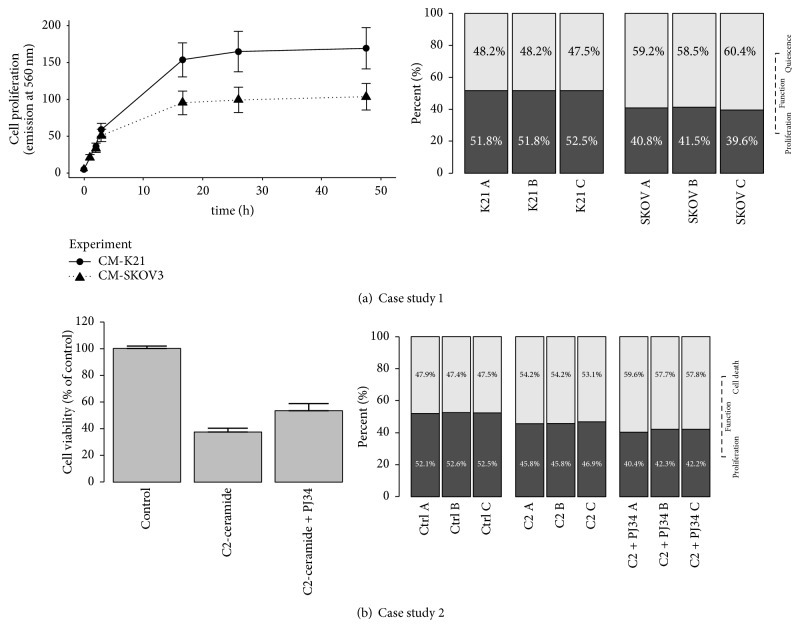
The comparison of the experimental results (left) with the theoretical estimation of functional proportions in subpopulations (right) for both analyzed case studies: (a) compares proliferation activity in the 48th hour of SKOV3 experiment; (b) compares behaviour in the 6th hour of the SH-SY5Y neuroblastoma experiment.

During the next phase, the obtained marker genes sets were used for ssKL method to predict the final proportion of different cell activities in the analyzed samples. In both case studies we observed significant differences in fractions of cells exhibiting investigated behaviours (see Figure 3).

In order to validate presented methods and significance of their outcomes we have performed the log-likelihood ratio test. It was measured how much the complex model with more degrees of freedom (assuming functionally heterogeneous population) fits the data better than the hypothesis assuming homogeneous population (*H*_0_). In each experimental condition *H*_0_ was rejected in favor of *H*_1_ (see Appendix C).

In most of the analyzed experimental variants our results were consistent with the results of biological assays and the literature based predictions. Our analysis indicate that secretion products of both SKOV3 cells and the fibroblast cell line K21 are able to activate cellular proliferation. However, the effect of stromal cells is much more pronounced. The results of predictions based on MPH method have been completely confirmed by Resazurin Reduction Assay.

Concerning the neuroblastoma cell line we have shown that in the control environment the fraction of proliferating cells is higher than in both experimental conditions, which was expected and consistent with MTT assay. Moreover, we observe that the reaction to external treatment is the strongest in the 6-hour time frame and seems to stabilize around 24th hour.

Interesting fact is related to our results concerning C2-ceramide and C2-ceramide + PJ34 experiments on the fraction of proliferating cells, since it varies from the results obtained using the MTT assay. Our computational method suggests that PARP inhibition along with C2-ceramide supplementation results in lower proliferation abilities than in experiment without the inhibition. This fact was not detected in our MTT assay probably due to its limitations, as well as in the analogous experiment presented in [13].

The predicted proportions for each sample are presented in Table 1. What is worth emphasizing is the behaviour of replicates for given time and setting which is stable (i.e., average standard deviation ≈ 0.01).

### 4.3. Functional Validation

The additional validation of our results was performed using the classical approach to the functional analysis of transcriptomic data. We intend to verify if profiles detected by application of the MPH method are consistent with general, average tendencies indicated by functional analysis tools. In order to identify molecular and cellular functions altered by treatments we performed core analysis using Ingenuity Pathway Analysis tool (IPA). The statistics for the most influenced functions reported in this section are listed in Table 2.

In the first case study we selected 3946 differentially expressed genes based on *p* value and cut off the number to 2238 based on the fold change. In the second case 3983 genes were selected for further analysis as distinguishing three experimental conditions, that is, control, C2-ceramide treated, and both C2-ceramide and PJ34 treated.

For the cells treated with fibroblast enriched medium (CM-K21) in the first case study, the top molecular and cellular function detected by* the disease and function* (IPA tool) was* cellular growth and proliferation* with 827 molecules involved. Within this cluster the highest activation was reported to* proliferation of cells* and* cell proliferation of carcinoma cell lines*. For the treatment with ovarian cancer enriched medium (CM-SKOV3), the most altered functions were* cell death and survival* and* cell cycle*, but in this case they were both reported as decreased. The representative clusters are* cell death* and* M phase*. The following top changed function was* cellular development* including downregulated clusters of* differentiations* and* senescence*. Clearly, the inhibition of cellular death, cell cycle, and differentiation indicate the cell quiescence.

In the second case study IPA analysis revealed significant enrichment in the* cellular growth and proliferation* in both C2-ceramide and C2 with PJ34 treated cells and this category was on the second position after* cellular movement* in C2 variant and* cellular development*. According to the *p* value in cells treated with C2 and PJ34* proliferation of tumor cell lines* was the first affected function and* proliferation of neuronal cells* was on the third position. In the C2-ceramide treated cells* proliferation of cells* was on the second position. It should be emphasized that in both variants proliferation scored very low *p* value what indicates high data enrichment. On the basis of *Z*-score its activation was decreased in comparison to control samples. According to activation *Z*-score in C2-ceramide treated cells the highest decrease was designated for the functions related to the* organization of cytoskeleton* and* cytoplasm*; however* proliferation of cancer cells* was the third decreased function. While increased activation was predicted for the functions as* morbidity or mortality* and* organismal death*,* apoptosis* was the fourth function. In the second experimental variant (C2-ceramide with PJ34) in the top of the relevant decreased functions were those related to cellular movement and development of neurons (e.g.,* formation of cellular protrusions, development of neurons*). However activation of* cell survival* and* proliferation of cells* was predicted to be decreased in comparison to control cells. What is more, in this experimental variant* necrosis*,* cell death,* and* apoptosis* were in the top of activated functions.

On the basis of the above findings, we conclude that the effect of the C2-ceramide enhanced by the PARP inhibition affects the ability of cells to divide and cellular viability as well as genetically controlled reorganization of cellular shape. This intriguing discovery should be subjected to the further analysis.

### 4.4. New Marker Genes

In the last step of the MPH method we selected new markers which characterize cellular subpopulations of our interest. New markers were selected according to the top differences in genes involvement in the functional expression profiles supported by biological knowledge. In the ovarian cancer case study the method revealed the two additional genes characteristic for the cellular quiescence: PI16 and WNT7A. Peptidase inhibitor 16 (PI16) was reported to inhibit cell migration and proliferation and determine cellular quiescence [14]. WNT7A maintains stemness [15, 16] and exerts an antiproliferative effect [17, 18]. The top distinctive genes for the proliferation metaprofile include, besides previously used markers, numerous genes related to malignancy and ovarian cancer progression, in particular cytokines and their receptors: CXCL1, IL1A, CXCL8, IL1B, IL1R1, and IL1R2 [19–22].

On the other hand neuroblastoma cells which entered the cell death pathway are characterized by activation of EGR1, VEGF, and GDF15. While the EGR1 is known to possess proapoptotic properties [23], VEGF and GDF15 are usually described as prosurvival proteins [24, 25]. Nevertheless, recently it was reported that overexpression of GDF15 (which is also observed in our experiment) induces apoptosis of breast cancer cells [26]. There is also some evidence that VEGF stimulates apoptosis through the ERK1/2 signaling pathway [27]. We believe that these three proteins provide unique markers for the ceramide induced death of the neural cancer cells.

Top genes characterizing each functional profile determined by the MPH method are listed in Appendix D along with their raw expression levels and fold changes.

## 5. Discussion

In this article, we propose a novel approach to the analysis of transcriptomic data from cellularly homogeneous samples aiming to describe their functional heterogeneity. The main innovation of our idea is based on the assumption that in a population of cells each individual cell conducts its own molecular function. Moreover, each cell in the population may respond in a different way to an external stimuli. These observations resulted in the development of the MPH method that estimates the proportion of cell subpopulations conducting specific cellular functions along with their transcriptomic description.

The above description of our results proves that our function characteristic profiles derived from the computational interpretation of transcriptomic data are functionally consistent. We have concluded that they actually correspond to the molecular subpopulations of both ovarian cancer and neuroblastoma cells that were studied.

*Ovarian Cancer Cell Line*. Based on the literature, both healthy fibroblasts (K21) and ovarian cancer cell line (SKOV3) were expected to boost cancer cells proliferation via paracrine manner. We found that conditioned media were sufficient to trigger ovarian cancer cell divisions regardless of serum depletion. Both* in vitro* experiments and microarray analysis prove that fibroblasts stimulate proliferation extensively, while ovarian cancer secreted factors exert a more subtle effect. This is consistent with the metaprofile results showing that percentage of proliferating cells is significantly higher in the culture with fibroblasts enriched medium. The* in vitro* experiments support the hypothesis that fibroblasts stimulate proliferation extensively, while ovarian cancer secreted factors exert a more subtle effect. These results are fully consistent with the conclusions based on the output provided by the MPH method. Based on the transcriptomic profiles we identified two cellular activities in the analyzed sample, that is, proliferation and quiescence, while the calculated proportions show that the percentage of proliferating cells is significantly higher in the culture with fibroblasts enriched medium.

The core analysis (IPA tool) of the significantly altered genes revealed the activation of cell cycle related genes in the CM-K21 condition. In parallel, in CM-SKOV3 treatment, we observe downregulation of differentiation, proliferation, senescence, and cell death suggesting cell dormancy. Although proliferation was clearly determined in both the first functional profile from the MPH algorithm and the raw transcriptomic data, the opposite process was not automatically defined. None of the commonly used programs (DAVID, IPA, Molgen) managed to identify dormancy or cell quiescence. However, 8 out of 12 of the most differentiating genes for the second functional profile were linked to quiescence in various studies. The MPH method requires manual analysis, but it allows more profound insight and identification of the nontrivial processes and corresponding markers.

In order to additionally verify the outcome of the MPH method for the ovarian cancer case study we have selected the third group of genes related to the cell signaling processes. It turned out that the final proportions are consistent with the two subpopulations case and the method predicts relatively low level of signaling activity (see Appendix E).

*Neuroblastoma Cell Line*. Similarly, in the second case study the MPH results support the C2-ceramide related hypotheses based on the literature. Both experimental and computational experiments confirm that in the presence of C2-ceramide the cell death processes increase their activity and the fraction of proliferating cells is lower than in the control sample. The same trends were detected between the control and second experimental condition (C2-ceramide + PJ34). The control sample manifests higher proliferation activities and repressed cell death processes. However, our computational analysis suggests that the PARP inhibitor (PJ34) does not increase the cell viability in populations supplied with the C2-ceramide. This fact is not consistent with the results from the MTT assay reported in [13].

However, since the functional analysis provided in the results section supports our computational results we suppose that the cause for this inconsistence may be the assay used to measure cells viability. The MTT assay depends on concentration and activity of NAD(P)H-dependent cellular enzymes [28], while PARP actively reduces the concentration of NAD(P)H [29]. We suspect that the discrepancy between the results of the MPH method and the MTT assay may result from PARP dependent changes in NAD(P)H balance or PARP related silencing of cellular oxidative stress or might have other unknown causes. We suggest further verification of PJ34 effect on ceramide induced cell death.

Summarizing, in both case studies the MPH method provides insightful interpretation of transcriptomic data, which is consistent with literature and functional analysis performed by IPA tools. However, in addition it expands our knowledge on the composition of the measured transcriptomic signal by the microarray experiments. We approximate the differences in gene expression levels for cells performing various molecular process and the proportions in which they are mixed.

The successful functional validation justifies the applicability of the proposed novel approach to the analysis of transcriptomic data retrieved from homogeneous samples. We illustrate that the methods, used previously at the cellular level to determine the input of each cell-type on the finally observed transcriptome, can be applied to the problem of a subcellular, regulatory nature. The method itself is stable and computationally efficient and provides statistically significant outcome.

Finally, it should be noted that methods that engage the nonnegative matrix factorization (NMF) techniques are also applicable in case of RNA-seq data analysis. Various modifications of NMF have been lately used for the inference from the RNA-seq results. For simple detection of the most differentiating genes between experimental and control ones, a method called discriminant NMF (DNMF) was suggested. The DNMF incorporates the Fisher's criterion into NMF by maximizing the distance among any samples from different experimental conditions, meanwhile minimizing the dispersion between any pairs of samples in the same class [30].

Additionally, the already presented DSection method was used by Dozmorov et al. [31] to describe the contribution of different cell types in systemic lupus erythematosus (SLE) pathogenesis where transcriptomic data were provided from RNA-seq experiments. Also, it should be mentioned that apart from the NMF there are other approaches to determination of cell-specific transcriptome, for example, quadratic programming approach suggested by Gong et al. [32]. Nevertheless, none of these techniques were used to attempt the subcellular task that we address in this paper. That is why we believe that the MPH approach not only is novel, but also highlights the fact that transcriptome analysis usually is based on multilevel assumptions and simplifications, which may obfuscate some subtle facts important in many biomedical aspects (e.g., diagnosis, drug resistance).

## 6. Conclusions

In this study we presented a novel methodological approach to quantify the functional heterogeneity of a homogeneous cell population based on transcriptomic data. To this aim we adopted the method proposed for quantification of cell proportions in heterogeneous tissue samples (e.g., mixed tissues) from expression data. Our model framework exploits the methodology designed for RNA expression microarrays applied for heterogeneous tissues. However, it should be emphasized that our novel approach can also be effectively applied to RNA-seq data by adapting the procedures proposed in [33, 34].

The presented case studies were focused on the effect of peritoneal paracrine signaling in ovarian cancer cells and the role of ceramide in mediating cell death in neuroblastoma cells. With the MPH method, in the ovarian cancer study, we identified two cellular activities, that is, proliferation and quiescence mixed in the proportion consistent with the experimental data. Our computational method also quantified the activity of C2-ceramide in time and its influence on cell metabolic activity in a consistent way with the MTT assay. Finally, the method allowed obtaining biologically relevant results describing the biologically meaningful interdependence between C2-ceramide and the PJ34 compound. In this case the specificity of molecular experiment requires the use of additional methods to verify the results of the MTT assay referring to cells viability.

Here, it should be mentioned that the presented framework could be further improved. Both DSection and ssKL procedures are characterized by the bias towards discrimination based mainly on fold changes in a given expression dataset. One should note that in the current implementation the ssKL method prevents the use of markers that differ on the direction of expression changes. The appropriate improvement of the decomposition method can constitute a very useful strategy for further research.

## Supplementary Material

AppendixC_Influence_of_marker_genes.xlsx: The file contains results for various group of genes to determine such properties as robustness of outcome and its correlation with the fold change.
AppendixD_Top_profiles.xlsx: The file contains lists of top 50 genes characterizing all functions from analyzed case studies.

## Figures and Tables

**Table:** (a) Case study 1

	K21			SKOV		
Proliferation	0.518	0.518	0.525	0.408	0.415	0.396
Quiescence	0.482	0.482	0.475	0.592	0.585	0.604

**Table:** (b) Case study 2

6 h	Control			C2–cer			C2–cer + PJ34		
Proliferation	0.521	0.526	0.525	0.458	0.458	0.469	0.404	0.423	0.422
Cell death	0.479	0.474	0.475	0.542	0.542	0.531	0.596	0.577	0.578

**Table 2:** Levels of statistical significance of each molecular function that was detected by IPA in each case study.

Molecular function	*Z*-score	*p* value
CM-K21		
Cell survival	3.264	1.08*E* − 22
Cell viability	3.196	3.84*E* − 20
Cell viability of tumor cell lines	3.312	1.63*E* − 11
Proliferation of carcinoma cell lines	1.292	1.86*E* − 07
Formation of cellular protrusions	−1.757	5.08*E* − 07

CM-SKOV3		
Interphase	−2.656	1.46*E* − 14
M phase	−1.789	2.42*E* − 12
Differentiation	−1.278	4.52*E* − 07
Senescence	−0.978	2.65*E* − 06
Assembly of cells	1.534	1.20*E* − 07
Cell death	−0.437	5.56*E* − 24
Necrosis	−0.712	2.65*E* − 23

C2		
Proliferation of cells	−2.520	4.97*E* − 11
Proliferation of cancer cells	−3.136	2.26*E* − 05
Apoptosis	2.039	2.07*E* − 04

C2 + PJ34		
Proliferation of tumor cell lines	−0.760	7.34*E* − 09
Proliferation of neuronal cells	−1.808	4.70*E* − 08
Cell survival	−2.661	1.87*E* − 03
Proliferation of cells	−2.417	1.77*E* − 06
Necrosis	2.482	1.34*E* − 04
Cell death	3.338	6.48*E* − 03
Apoptosis	3.777	7.44*E* − 04

**Table 3:** List of the functional marker genes that were used in the first case study.

Symbol	Name
Proliferation	
CDC20	Cell Division Cycle 20
TK1	Thymidine Kinase 1
KNL1	Kinetochore Scaffold 1
CENPE	Centromere Protein E
STIL	SCL/TAL1 Interrupting Locus
ANLN	Anillin Actin Binding Protein
NDC1	NDC1 Transmembrane Nucleoporin
NUF2	Cell Division Cycle-Associated Protein 1
KIF20A	Kinesin Family Member 20A
PLK4	Polo Like Kinase 4
CCNB1	Cyclin B1
CCNA2	Cyclin A2

Quiescence	
COL5A1	Collagen Type V Alpha 1 Chain
TGFBI	Transforming Growth Factor Beta Induced
TCEA2	Transcription Elongation Factor A2
WNT9A	Wnt Family Member 9A
MMP11	Matrix Metallopeptidase 11
LAMB1	Laminin Subunit Beta 1
KRT14	Keratin 14
LTBP1	Latent Transforming Growth Factor Beta Binding Protein 1
PHLDB1	Pleckstrin Homology Like Domain Family B Member 1
TIMP3	TIMP Metallopeptidase Inhibitor 3
LRP1	LDL Receptor Related Protein 1
COL18A1	Collagen Type XVIII Alpha 1 Chain

**Table 4:** List of functional marker genes that were used in the second case study.

Symbol	Name
Cell death	
DDIT3	DNA Damage Inducible Transcript 3
ERN1	Endoplasmic Reticulum To Nucleus Signaling 1
JUN	Jun Proto-Oncogene, AP-1 Transcription Factor Subunit
SQSTM1	Sequestosome 1
SMPD1	Sphingomyelin Phosphodiesterase 1
TGFB1	Transforming Growth Factor Beta 1
PRNP	Prion Protein
CEBPB	CCAAT/Enhancer Binding Protein Beta
NQO1	NAD(P)H Quinone Dehydrogenase 1
NR4A1	Nuclear Receptor Subfamily 4 Group A Member 1
CTSB	Cathepsin B
ZMAT3	Zinc Finger Matrin-Type 3

Proliferation	
CD24	CD24 Molecule
NRP1	Neuropilin 1
TNS3	Tensin 3
MYC	V-Myc Avian Myelocytomatosis Viral Oncogene Homolog
CD38	CD38 Molecule
CCNA2	Cyclin A2
FGF7	Fibroblast Growth Factor 7
MST1R	Macrophage Stimulating 1 Receptor
MYCN	V-Myc Avian Myelocytomatosis Viral Oncogene Neuroblastoma Derived Homolog
ETS1	ETS Proto-Oncogene 1, Transcription Factor
EDNRA	Endothelin Receptor Type A

**Table 5:** 

Cell signaling	
Symbol	Name
POSTN	Periostin
ABCA1	ATP Binding Cassette Subfamily A Member 1
HMGA1	High Mobility Group Protein A1
IL1B	Interleukin 1 Beta
DDR2	Discoidin Domain Receptor Tyrosine Kinase 2
CEBPB	CCAAT/Enhancer Binding Protein Beta
YARS	Tyrosyl-TRNA Synthetase

**Table 6:** 

	K21			SKOV		
Proliferation	0.4549	0.4544	0.4571	0.4105	0.4140	0.4057
Quiescence	0.4531	0.4537	0.4509	0.4972	0.4938	0.5021
Signaling	0.0920	0.0919	0.0920	0.0923	0.0921	0.0922

## Appendix

### A. Biological Background

#### A.1. Case Study 1: Peritoneal Microenvironment

This study focuses on the microenvironmental effect of the peritoneal cavity on prometastatic properties of ovarian cancer cells. Progression of malignant tumors does not depend exclusively on autonomous properties of the cancer cells but is deeply influenced by tumor stroma reactivity and undergoes a strict microenvironmental control [35]. Ovarian tumors heavily contribute to the peritoneal microenvironment and affect floating cancer cells in both autocrine and paracrine manner. Stroma cells enhance carcinomatosis of many kinds of cancer by promoting the epithelial-mesenchymal transition, a phenotypic switch resulting in enhanced tumor cell motility and invasiveness, increased metastatic propensity, and resistance to chemotherapy [35, 36]. Ovarian stromal cell-type is critical in determining whether metastasis occurs [21]. It was observed that fibroblasts derived from the omentum, the richly vascularized fatty subperitoneal layer draping the ovaries, augmented ovarian cancer cell adhesion and invasive behaviour [37, 38], whereas omentum-derived mesothelial cells functionally inhibited ovarian cancer cell aggressiveness [37]. We scrutinized the influence of secretion of the two kinds of cells, fibroblasts and ovarian cancer cells, which are known for their stimulating paracrine activity.

We cultured epithelial ovarian cancer cell line SKOV3 with media enriched by secretion products of the SKOV3 cells and the healthy fibroblast cell line K21. The treatment alternates transcriptomes and biological properties of the cells in regard to proliferation, migration, chemoresistance, and anoikis survival. The output of our research will be described within a separate publication.

#### A.2. Case Study 2: C2-Ceramide and PARP Inhibition

Microarray experiment was designed to study the role of ceramide, bioactive sphingolipid in cancer signaling [39]. In particular, we studied the influence of C2-ceramide and PJ34, which is the inhibitor of PARP on viability of the neuroblastoma SH-SY5Y cell line. According to the literature we expected C2-ceramide to stimulate the cell death and suppress the cell proliferation [40, 41]. The role of PARP in this process was reported in [13]. However, the literature reports on the influence of PARP inhibition on the cell viability are contradictory [42, 43]. PARP1 is an abundant nuclear enzyme. It belongs to nucleosome-binding architectural proteins that promote structural alterations in chromatin and modulate transcriptional responses [44–46]. PARP1 catalyzes the polymerization of ADP-ribose units from donor NAD^+^ molecules on target proteins involved in DNA repair [47]. The activation of PARP1 was observed during necrotic cell death [48, 49]. What is more, the inhibition of this enzyme is known to switch the cell death from the necrotic mode to the apoptotic one, which might be related to the availability of cellular reductive power (NAD^+^) [42]. PARP was also reported to induce caspase independent cell death called parthanatos [50–52].

Although the molecular basis of C2-ceramide and PARP1 induced cell death remain vague, it is not in the scope of this paper to explain the biological principles of this process. With this paper our main aim is to present a novel computational method for transcriptome analysis of homogeneous samples, to get a quantitative insight into ongoing molecular processes.

### B. Marker Genes Characteristics

Tables 3 and 4 contain full names of genes composing functional fingerprints used in the second stage of the MPH method.

### C. Statistical Significance of Results

The log-likelihood ratio test was performed to statistically validate our outcome. We decided to perform the validation only for the larger dataset, that is, the second case study. It was measured how much the complex model with more degrees of freedom (assuming heterogeneous population) fits the data better than the hypothesis assuming homogeneous population (*H*_0_). Namely, assuming that *X* is a vector of gene expression levels our null and alternative hypotheses are *H*_0_: *X* ~ *𝒩*(*μ*_1_, *σ*_1_^2^) and *H*_1_: *X* ~ *p* · *𝒩*(*μ*_1_, *σ*_1_^2^)+(1 − *p*) · *𝒩*(*μ*_2_, *σ*_2_^2^).

We used the Expectation-Maximization (EM) algorithm to estimate the best parameters and calculate the likelihood for both models.

Since there are only 3 measurements per experimental condition, statistical confirmation of our results is hard to provide (i.e., on three samples there is no basis for rejection of null hypothesis: sample represents unimodal normal distribution). Nevertheless, in order to provide at least partial statistical justification of our results, using *k*-means algorithm, for each experimental condition we have clustered all genes that were used in the analysis. Such clustering provided larger samples with common expression pattern that could have been analyzed with EM algorithm. An effective size of the sample for EM algorithm was set to 100, determining the number of expected clusters in *k*-means equal to 40.

In each experimental condition for most of the clusters the likelihood ratio test rejected *H*_0_ in favor of the *H*_1_. For C2-supplied experiment *M*_0_ was rejected 37 (with mean *p* value: 6.6 · 10^−4^), 39 (1.2 · 10^−3^), and 39 (4.4 · 10^−4^) times (out of 40 cases) for 3rd, 6th, and 24th hour of experiment, respectively. Similarly, for the above time points in PJ34 + C2-supplied experiment *M*_0_ was rejected in 36 (3.8 · 10^−4^), 39 (3.4 · 10^−4^), and 38 (8.0 · 10^−4^) cases and for control samples in 38 (8.2 · 10^−4^), 39 (1.0 · 10^−3^), and 39 (1.5 · 10^−3^).

In case of C2-supplied experiment the hypothesis of the *M*_0_ model was rejected in favor of the *M*_1_ model in 798 (3 h of experiment), 824 (6 h), and 759 (24 h) out of 1000 cases, with mean *p* values 4.244 · 10^−3^, 3.883 · 10^−3^, 5.720 · 10^−3^ in respective time points.

Same verification procedure was performed for the control samples and PJ34 + C2-supplied experiments, all resulting in 751 up to 811 rejected *M*_0_ models in all time points, with mean *p* values from (4.224 · 10^−3^, 4.851 · 10^−3^) interval.

These results suggest that, indeed, in each experimental condition, it was reasonable to expect that the observed expression level is a composition of signals from different sources.

*Marker Genes and Proportions Prediction*. AppendixC_Influence_of_marker_genes.xlsx file contains results for various group of genes to determine such properties as robustness of outcome and its correlation with the fold change.

### D. Functional Profiles Characteristics

The file AppendixD_Top_profiles.xlsx contains lists of top 50 genes characterizing all functions from analyzed case studies.

### E. Three Subpopulations' Detection in the Ovarian Cancer Case Study

For the ovarian cancer case study we have selected an additional group of genes related to the cell signaling processes as shown in Table 5.

Considering the same groups of genes for proliferation and quiescence processes as before, we have obtained the following preliminary results from the MPH method as shown in Table 6.

The main observation here is the fact that these proportions are consistent with the main result from the manuscript for two subpopulations; that is, in K21 samples the proliferation activity is higher. Next, when it comes to the signaling processes it is of low percentage and at the similar level in each of the six samples, which means that it does not necessarily differentiate the analyzed samples and is of quite low importance in the context of our study. However, it should be noted that this process is quite general and thus has nonempty molecular overlap with other functional subpopulations. In our case, when functional subpopulations are defined it is the best practice to keep then mutually exclusive if possible. Nevertheless, we believe that the consistency fact additionally proves that considering two processes in our case studies was justified.
